# SARS-CoV-2 host prediction based on virus-host genetic features

**DOI:** 10.1038/s41598-022-08350-6

**Published:** 2022-03-17

**Authors:** Irina Yuri Kawashima, Maria Claudia Negret Lopez, Marielton dos Passos Cunha, Ronaldo Fumio Hashimoto

**Affiliations:** 1 Institute of Mathematics and Statistics, University of Sao Paulo, São Paulo, 05508-090 Brazil; 2 Scientific Platform Pasteur USP, University of Sao Paulo, São Paulo, 05508-020 Brazil

**Keywords:** Machine learning, SARS-CoV-2

## Abstract

The genetic diversity of the Coronaviruses gives them different biological abilities, such as infect different cells and/or organisms, a wide spectrum of clinical manifestations, their different routes of dispersion, and viral transmission in a specific host. In recent decades, different Coronaviruses have emerged that are highly adapted for humans and causing serious diseases, leaving their host of unknown origin. The viral genome information is particularly important to enable the recognition of patterns linked to their biological characteristics, such as the specificity in the host-parasite relationship. Here, based on a previously computational tool, the Seq2Hosts, we developed a novel approach which uses new variables obtained from the frequency of spike-Coronaviruses codons, the Relative Synonymous Codon Usage (RSCU) to shed new light on the molecular mechanisms involved in the severe acute respiratory syndrome coronavirus 2 (SARS-CoV-2) host specificity. By using the RSCU obtained from nucleotide sequences before the SARS-CoV-2 pandemic, we assessed the possibility of know the hosts capable to be infected by these new emerging species, which was first identified infecting humans during 2019 in Wuhan, China. According to the model trained and validated using sequences available before the pandemic, bats are the most likely the natural host to the SARS-CoV-2 infection, as previously suggested in other studies that searched for the host viral origin.

## Introduction

The first reported case of Coronavirus Disease 2019 (COVID-19) occurred in the city of Wuhan, Hubei province, China, in late December 2019^1,2^. The viral agent associated with this new pneumonia — The Severe Acute Respiratory Syndrome associated with Coronavirus 2 (SARS-CoV-2) (*Sarbecovirus* subgenus, *Betacoronavirus* genus) — quickly spread worldwide causing a pandemic with global impact^3^. SARS-CoV-2 represents the seventh know coronavirus to circulate in a human-to-human transmission chain of the *Coronaviridae* family. While the SARS-CoV, MERS-CoV, and SARS-CoV-2 can cause severe disease, the HKU1, NL63, OC43, and 229E cause mild symptoms, and for all of them, their ancestral hosts are not human^4^.

Viruses genetically related to SARS-CoV-2 have been found in bats^5^ and pangolins (*Manis javanica*)^6,7^ in the Asian continent. The divergence of the SARS-CoV-2 from related viruses represents decades of evolution^8^, which makes it difficult to suggest which is the probable host involved in the SARS-CoV-2 emergence. Although the biological mechanism responsible for viruses spillover remains uncertain^9,10^, since its first description, this virus has shown sustained transmission in the human-to-human transmission chain without adaptive genomic changes^11^, demonstrating its pandemic potential.

The Coronaviruses are a group of viruses that differ from each other by their genomic characteristics and their ability to infect different groups of organisms^12^. They are members of the subfamily *Coronavirinae* in the family *Coronaviridae* and the order *Nidovirales*, according to International Committee on Taxonomy of Viruses. Studies have shown that these viruses can have abnormally high replication fidelity^13^, having in their genomic structure, a set of RNA processing enzymes that have improved the low fidelity of RNA replication^14^. Despite having important genomic conservation at apical levels of their phylogenetic structure, they present considerable genetic differences at basal levels, this difference being important for studies that explore levels of adaptation to different hosts^12^. The discriminative characteristics that make it possible to distinguish SARS-CoV-2 from other related coronaviruses (SC2r-CoVs) to it are concentrated mainly in the Spike gene, which appears to be the main component of the virus associated with host specificity^15^.

Genomic characteristics of the SARS-CoV-2 spike gene are known to be linked to its ability to infect humans^16^. Recent studies have shown descriptive results on the genomic composition of SARS-CoV-2, comparing them to other coronaviruses^17–19^, indicating that this virus has shared discriminating characteristics when compared to its homologs. Sequences coding to the Spike protein were previously used to build a computational tool for inference of potential hosts using genomic data^12^, such as mononucleotide and dinucleotide composition.

In a recent work, researchers concluded that the use of features based on genomic composition can better predict the risk of a virus infecting humans than those based on phylogenetic distance^20^. In our study, we applied a new approach using the Relative Synonymous Codon Usage (RSCU) to training and validate the seq2hosts tool developed by Tang et al.^12^ and we complemented this previous study using the recent information and machine learning techniques as Principal Component Analysis (PCA) and Mahalanobis distance (MD). For this purpose, we use this classifier to find the MD of new coronaviruses sequences and host prototypes, suggesting other possible hosts.

## Results

### Phylogenetic analysis, dimensionality reduction, and model training

**Figure 1:**
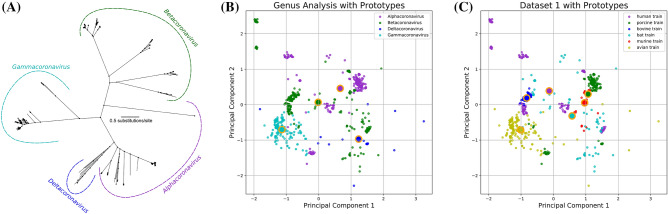
Characterization of the training dataset (Dataset 1). (**A**) Phylogenetic characterization estimated based on maximum likelihood, and showing all the Coronaviruses genus. The tree was generated with IQ-TREE 1.5.5^21^ available at http://www.iqtree.org and visualized with FigTree 1.4.4^22^ available at http://tree.bio.ed.ac.uk/software/figtree/; (**B**) Two Dimensional PCA reduction with prototypes according to the different Coronaviruses genus; and (**C**) Two Dimensional PCA reduction with prototypes according to the different primary host. The different colours in (**B**) and (**C**) represents each group of genus or host class using the training data set (Dataset 1). For some hosts or even between the genus, we can observe some clouds of points concentrated, while in other conditions, as in bats, the samples are scattered in different positions of the graph.

The phylogenetic analysis based in the aligned aminoacid sequences indicated that the sequences are grouped into four large groups, characterized as Coronaviruses genera, in the *Alphacoronavirus*, *Betacoronavirus*, *Deltacoronavirus*, and *Gammacoronavirus* (Fig. 1A). The reduction of the 59 features (obtained from the genome through RSCU codification) into two-dimension space with Principal Component Analysis (PCA) enables us to visualize a scatter plot of the first two dimensions of Dataset 1 (Fig. 1B,C) containing all samples of each host together with their prototypes (centroids). In general, the points of some viral species, such as bovine and murine (blue and red colors, respectively) concentrates in one specific point cloud, suggesting that contains relevant information about the host specificity, but in other cases, such as to the human and bats, the points are spaced in space.

### Validation

**Figure 2:**
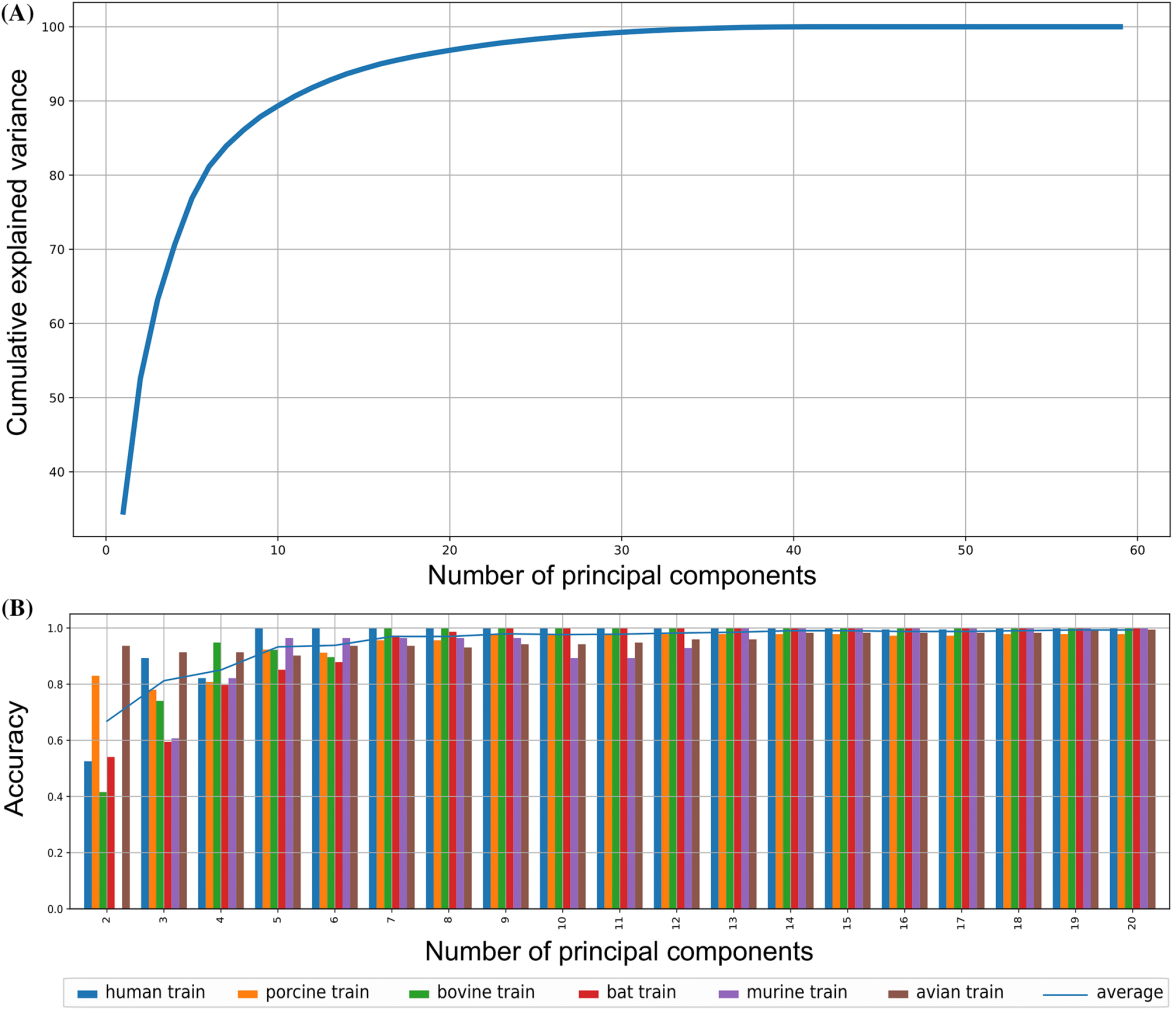
Classifier performance. (**A**) Cumulative explained variance using distinct number of principal components; and (**B**) Accuracy to each host using different number of principal components. From 20 PCs onwards there is no longer significant increase in accuracy. The Confusion Matrix used to obtain this diagram can be found in Supplementary Table S2.

The validation of rating performance was obtained using the Dataset-1 and indicated a progressive increase in accuracy with an increase in the number of components retrieved by the PCA calculation (Fig. 2). With 2 components the total explained variance obtained was 0.52593 with accuracy of prediction 0.668493 whereas with 20 components variance and accuracy increased to 0.9683 and 0.993151, respectively, so the prediction of the sequences from the remaining datasets was computed with the 20-dimensional projection distances measures. The confusion matrix for each dimension reduction from 2 to 20 can be found in Supplementary Table S2.

### Host predictions for viruses suspected of involving transmission between species

The trained model was applied to 47 additional sequences (Dataset-2) that we only listed the hosts from which they were isolated, with all the samples with evidence of potential transmission between species, as used previously^12^. To the sequences isolated from palm civets, here called Civets-CoVs (SARS-CoV), the predicted host was humans. According to Tang et al.^12^, bats and humans are the hosts of these viral strains, but bats are the preferable hosts; the porcine-CoV, was a SARS-associated coronavirus that was transmitted from human to porcine, and the prediction results indicated the same results obtained to the dromedary-CoVs, with bats as the predicted host; the SARS-like coronaviruses sequences isolated from bats, the results obtained using MD correctly confirmed the first host as bats, followed by humans; to the sequences isolated from Dromedary, the Dromedary-CoVs (MERS-CoV), obtained after the outbreak in the Middle East in 2012 indicated that humans and bats are the first and second host, respectively and is corroborated by the first study^12^. The MD distances to the Coronavirus sample isolated from Alpaca (Alpaca-CoVs) indicated that the predicted host was bovine; surprisingly, the to the bovine-CoV (Human enteric coronavirus isolated from bovine) result, the predicted host was bovine; and to the Human-CoV (Human enteric coronavirus isolated from human) sequence, the predicted host was bovine (Table 1).

**Table 1:** Sequences used to the biological validation and prediction results for Datasets 2, 3 and 4.

Number of sequences	Isolation source	Natural host	Predicted host	Coronavirus specie	Dataset
30	Civet	Human	Human	SARS-CoV-1	2
1	Raccoon	Human	Human	SARS-CoV-1	2
9	Dromedary	Human	Human	MERS-CoV	2
1	Porcine	Human	Human	SARS-CoV-1	2
3	Bat	Bat	Bat	Bat SARS-like	2
1	Alpaca	Bovine	Bovine	Bovine Coronavirus	2
1	Human	Human	Bovine	Human enteric coronavirus	2
1	Bovine	Human	Bovine	Human enteric coronavirus	2
86	Human	Human	Bat	SARS-CoV-2	3
5	Bats or pangolin	Bat	Bat	SC2r-CoVs	4

### SARS-CoV-2 host prediction

**Figure 3:**
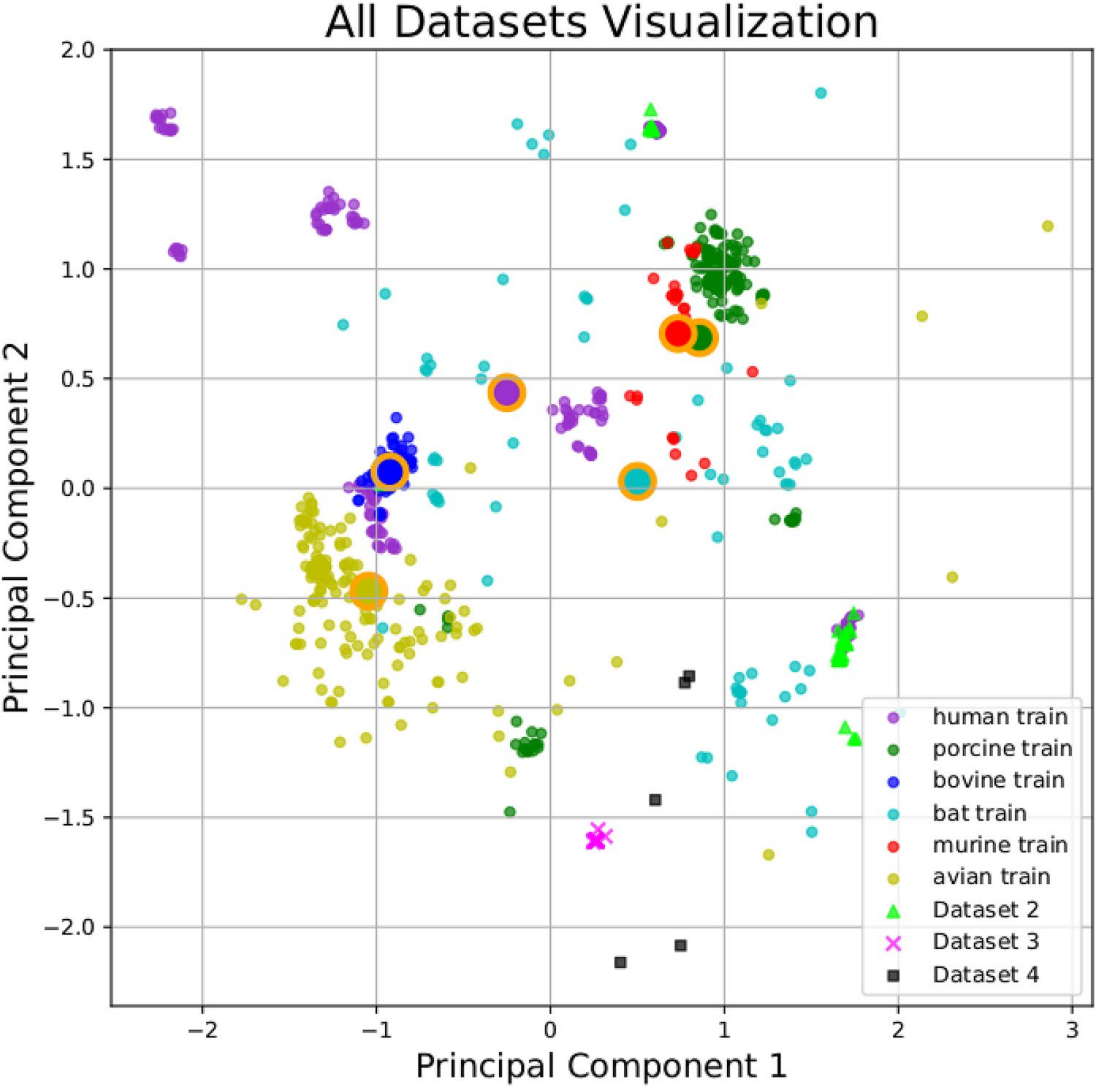
PCA reduction, all datasets. Dataset-1, training dataset; Dataset-2, testing; Dataset-3, SARS-CoV-2; Dataset-4, Bat Coronavirus, HCoV and Pangolin Coronavirus. Despite the Dataset-4 sequences being phylogenetically close to the Dataset-3 SARS-CoV-2 sequences^15^, we can notice that all of them do not cluster together when using RSCU as feature. Both Dataset-3 and 4 sequences were classified by our model as closer to bat coronaviruses than human coronaviruses.

To predict the SARS-CoV-2 host-associated, we used the classifier constructed and validated using MD and the Spike nucleotide sequences of the SARS-CoV-2 obtained from GenBank (Dataset-3). The predicted host was bat although samples were taken from humans (Table 1). The phylogenetically nearest sequencies to SARS-CoV-2 (SC2r-CoV), Dataset-4 were predicted to be hosted by bats. This result is completely congruent with the scientific evidence raised so far, which found SC2r-CoVs viruses in bats. The other tested hosts were not involved in this hosting system according to the prediction. Figure 3 presents all datasets in a two dimensional PCA scatter plot.

## Discussion

Since the beginning of the pandemic, the origin of SARS-CoV-2 has been an object of interest to the global scientific community, with several scientific initiatives carried out and even, with the World Health Organization (WHO) moving a mission as part of the One Health approach, to identify the zoonotic source of the virus and routes of introduction to the human circulation, including the possible role of intermediate hosts. Genomic comparisons of SC2r-CoVs and pandemic SARS-CoV-2 sequences suggested that the virus has required little to no significant adaptation to start a circulation in humans at the early phase of the pandemic^11^. The structural changes present in the SARS-CoV-2 when compared to SC2r-CoVs, which may be associated with a high capacity to infect human cells, are in the most divergent genome region, that codded to the Spike protein^16^. Two of the key changes that occurs in the pandemic virus are the specific receptor binding domain sequence and the inserted furin cleavage site^8,16^. However, we also know that specific nucleotide differences may be reflected in specific pattern of the codon usage for different SARS-CoV-2 genes, including the Spike gene, when compared to other coronaviruses^17^.

Throughout the natural course of coronavirus evolution, virus genomes accumulate mutations during their propagation. The underlying principle is that virus-host genetic information can provide the variables involved in the host infection specificity process, a valuable tool to explore the taxonomic classification of new viruses, track transmission chains, and the prediction of hosts that are likely to be involved in their replication cycles^12^. Recently, an approach using dual statistical models based on mono- and dinucleotide composition was used to predict the probable hosts for the Coronaviruses^12^. The results retrieved using a new group of sequences, the SARS-CoV-2 spike gene sequences (Dataset-3) as input for host prediction using the previous approach (Seq2Hosts platform)^12^, indicated that organisms predicted as hosts are avian and bovine for first and second prediction using the Mahalanobis Distance technique (April/2020). During the beginning of the pandemic, all the SARS-CoV-2 sequences exhibited low genetic diversity^23^. Further, the current evidence suggests that this inferenced result is not consistent with recent studies exploring evolutionary and biological aspects related to the virus, which show that both groups of organisms are not involved in the zoonotic circulation of SARS-CoV-2-related viruses, as well as SARS-CoV-2 has not yet been found in these organisms. The hosts known to be involved in viral circulation are mainly bats (in a zoonotic environment) and humans, although there is still a discussion about the intermediate host in this emergence process^5–7^.

To explore the same approach^12^, but now refining it to be precise in identifying hosts with a description known and sustained in the literature, we used a new group of features associated with virus-host specificity, the RSCU signature, which is a method to calculate the relative frequences of occurrence of the synonymous codons for each amino acid^24,25^. Beyond that, although the virus-host association can be recovered from nucleotide composition^26^, such as the RSCU used here, the codon usage is not restricted to adaptation to a host, but is critical for several biological processes, which reflect the combination of multiple selection and mutational pressure, which are critical for efficient transcription, nuclear export of virus RNA, tolerance to translation errors, and immune evasion^27,28^.

Interestingly, the PCA plot demonstrating the first two components, bat-related Coronaviruses are widely dispersed across the plot, with no sign of a cloud densely populated by many sequences. On the other hand, the Coronaviruses associated with humans are dispersed throughout the graph, however, with dense clouds of sequences in specific locations, as observed for the other Coronaviruses associated with other hosts. This result suggests that there is a high genetic diversity of Coronaviruses circulating among bats^29^, acting as key hosts of zoonotic coronaviruses^30^, and that, most likely, the circulation in other organisms is associated with viral spillover events from bats to other hosts. The results obtained using our proposed classifier based on the RSCU features indicated that the main group of organisms that are among those tested (avian, bat, bovine, human, murine, and porcine) which are involved as natural hosts for SARS-CoV-2 are bats based in the MD of the SARS-CoV-2 sequences to the prototypes. If so, these findings suggested that the RSCU can reflect biological meaning in terms of coronavirus adaptation to the cellular host machinery.

According to a recent study, the SARS-CoV-2 Spike protein could have a high probability of binding with the angiotensin-converting enzyme 2 (ACE2) receptors in rats, sheep, camels, and squirrels^31^. Also, for predicting the host tropism, the major ACE2 residues involved in the recognition of Spike protein of SARS-CoV-2, it was developed a homology-based model which analysis found that apart from humans, other animal species, like African green monkey, orangutan, dog, cat, tiger, cattle and pig exhibit the key residues, making these species likely susceptible hosts for SARS-CoV-2 virus attachment^18^. Recently, the joint international team WHO-China study concluded that the most closely related genomic sequences to the SARS-CoV-2 have been found in bats^32^.

Beyond the prediction results obtained using the RSCU calculations based in the MD, our study suggests that SARS-CoV-2 emerged to humans from bats. Furthermore, the high susceptibility and permissivity of mink and cats to the SARS-CoV-2 suggest that additional species of animals may act as a potential reservoir to the virus^33,34^. This new approach can be used to the: (*i*) description of the natural host, and the viral emergency host (viral spillover) in new Coronavirus emergence; (*ii*) it may be important to suggest new experimental models that are biologically close to natural hosts; (*iii*) it can be useful to study new coronaviruses with emergency potentials.

## Methods

### Datasets

This study was conducted based on the datasets previously described^12^. First, to the training tool, we used the same dataset used in the work previously developed^12^, with 730 sequences corresponding to the Spike gene of different viral specimens of coronavirus (Dataset-1), 196 of which belong to the human host, 182 to the porcine, 173 to the avian, 77 to the bovine, 74 to the bat and 28 to the murine. The model was tested using sequences that are not in the training data, we also used the same dataset provided previously by the authors as a test set (Dataset-2) corresponding to 47 spike gene sequences of SARS coronavirus — 30 collected from civets, 1 from raccoon and 1 from porcine, 9 Middle East respiratory syndrome coronavirus from dromedary, 3 Bat SARS-like coronavirus from bats, 1 Bovine coronavirus isolated from Alpaca and 2 Human enteric coronavirus — 1 from human and 1 from bovine^12^. The model was then applied for the prediction of the viral hosts involved in the viral replication biology of SARS-CoV-2, using 86 virus sequences retrieved from GenBank at 04-26-2020, all of them isolated from human samples (Dataset-3) and additionally 5 sequences of SARS-CoV-2 related viruses isolated from bats and pangolins (Dataset-4). Information about genbank accession number and species description of all data sets can be found in the Supplementary Table S1.

### Phylogenetic analysis

The phylogenetic inference was performed with a previously curated dataset (dataset-1)^12^. A phylogenetic tree was reconstructed based on aminocid Spike gene sequences using the Maximum Likelihood (ML) method implemented in IQ-TREE 1.5.5^21^ with automatic model selection by ModelFinder and using the Bayesian Information Criterion (BIC)^35^, which was the model of substitution: WAG+F+R7. The robustness of the groupings observed was assessed using an ultrafast bootstrap approximation (UFboot) during 1,000 replicates. The ML tree was visualized and plotted using FigTree v.1.4.4^22^. Taxon labels for sequences used in this work had the format: accession number/Coronavirus genera/Primary host.

### RSCU calculation

The codon is a combination of three nucleotides that encodes for an amino acid or a stop signal. Despite of there are 64 combinations of three nucleotides, considering that there are four nucleotides for the coding purpose, the number of amino acids are 20, because some amino acids can be coded by more than one triplet of nucleotide. So the genetic code is redundant, and it is described “degenerate”, that is, multiple synonymous codons refer to the same amino acid. For example, the codons GCU, GCC, GCA and GCG code the same amino acid Alanine (*Ala*).

The usage of synonymous codon for each amino acid is not random, it depends on the abundance of the respective tRNA of the organism. Selective pressure contributes to optimize gene expression inducing a bias to the presence of codons related to more abundant tRNA species^24^.

The expected number of occurrences of codon usage $E_i$ for a given amino acid *i* can be computed counting the number of that codon in the sequence normalizing it by the number of codons that code the same amino acid:

$$\begin{aligned} E_i = \frac{1}{n_i}\sum _{j=1}^{n_i}F_j^{(i)}, \end{aligned}$$

where $n_i$ is the number of synonymous codons for amino acid *i* ($1 \leqslant n_i \leqslant 6$) and $F_j^{(i)}$ is the number of occurrences of codon *j* for amino acid *i*. For example, for the amino acid Alanine (*Ala*), the number of synonymous codons is $n_{Ala}=4$, whereas $F_{GCU}^{(Ala)}$, $F_{GCC}^{(Ala)}$, $F_{GCA}^{(Ala)}$, $F_{GCG}^{(Ala)}$ are the frequencies of its synonymous codons.

So, in the case of Alanine, its expected number of occurrences of codon usage is $E_{Ala}=\frac{1}{4}\left( F_{GCU}^{(Ala)}+F_{GCC}^{(Ala)}+F_{GCA}^{(Ala)}+F_{GCG}^{(Ala)}\right)$.

The bias of each codon for a given amino acid that can be coded by more than one codon is estimated by calculating the Relative Synonymous Codon Usage $RSCU_j^{(i)}$ that is the number of times the codon *j* appears within a gene divided by the expected number of occurrences of its synonymous codons for the *i*th amino acid:

$$\begin{aligned} RSCU_j^{(i)} = \frac{F_j^{(i)}}{E_i}, \end{aligned}$$

where $F_j^{(i)}$ is the number of occurrences of the *j*th codon for the *i*th amino acid, which is encoded by $n_i$ synonymous codons^24^.

In the case of Alanine:

$$\begin{aligned} RSCU_{GCU}^{(Ala)}=\frac{F_{GCU}^{(Ala)}}{E_{Ala}} \quad \text {;} \quad RSCU_{GCC}^{(Ala)}=\frac{F_{GCC}^{(Ala)}}{E_{Ala}} \quad \text {;} \quad RSCU_{GCA}^{(Ala)}=\frac{F_{GCA}^{(Ala)}}{E_{Ala}} \quad \text {and} \quad RSCU_{GCG}^{(Ala)}=\frac{F_{GCG}^{(Ala)}}{E_{Ala}}. \end{aligned}$$

The values for UGG and AUG that codes respectively for Tryptophan and Methionine are always 1.0, since there is only one codon for them. In addition, the three stop codons can be excluded from the analysis, since they do not correspond to a tRNA. Computing the RSCU for valid codons for each sequence results in a 59 dimension feature space.

Viruses depend on the host machinery to replicate themselves and translate their proteins. As they require available tRNAs, they have the tendency to evolve a codon usage preference for the amino acid that can be coded by more than one codon, as closer as possible to their host. Therefore, the efficiency of the viral proteins production can be established selectively on genetic material mutation^36^. In this way, we hope that the RSCU can identify how close the viral species are to each host specified in the training data set.

### Dimensionality reduction

Our model consists of using the nearest prototype classifier with Mahalanobis distance after the PCA reduction in the feature table with the RSCU values.

Principal components analysis (PCA) is a popular approach for deriving a low-dimensional set of features from a large set of variables. Its is widely used in Bioinformatics to analysis of genome data and gene expression levels ^37^.

The goal of PCA is to find the directions of maximum variance in high-dimensional data and projects (by a linear transformation) it onto a new subspace with equal or fewer dimensions than the original one. The orthogonal axes, called “principal components”, of the new subspace can be interpreted as the directions of maximum variance given the constraint that the new feature axes are uncorrelated to each other ^38^.

In our case, we used PCA to reduce features dimensions from 59-dimension to smaller dimensional spaces starting with 2 components and increasing them. In order to visually explore the data, we used 2 components to plot the graphs presented in this text, Principal Component 1 (*PC*1) at the vertical axis $x_1$ and Principal Component 2 (*PC*2) at the horizontal axis $x_2$.

### The nearest prototype classifier

In a supervised classification, the *k*-nearest neighbor (KNN), or even the nearest neighbor (when $k=1$), can be used to classify an unknown sample, assuming that it will have characteristics similar to that of the neighborhood. The method consists of computing the distance to all the known samples and check the label of the *k* (usually an odd number to avoid ties) closest ones. The unknown sample is labeled by the majority of votes from its *k* neighbours.In this study, instead of using the KNN classifier, we consider using the nearest prototype classifier.

In the training dataset, there are six labels (avian, bat, bovine, human, murine, and porcine), corresponding to the hosts of viral species, each one encompassing a certain amount of samples.

We define the prototype of each label as the mean point in the feature space of all species that infect the same host. For example, reducing the feature space to two dimensions $x_1$ and $x_2$ axis, we can find the prototype in the center of the points that belong to the same host computing the average for these features for each host:

$$\begin{aligned} \mathbf {c_{host}} = (\bar{x_1}_{host},\bar{x_2}_{host}), \end{aligned}$$

where $\bar{x_1}_{host}$ is the average of $x_1$ values on the vertical axis and $\bar{x_2}_{host}$ is the average of $x_2$ values on the horizontal axis for a given host. Note that $\mathbf {c_{host}}$ after the PCA reduction is a point with two coordinates $\mathbf {c_{host}}=(c_1,c_2)$, and there will be six prototypes in our training data set: $\mathbf {c_{avian}}$, $\mathbf {c_{bat}}$, $\mathbf {c_{bovine}}$, $\mathbf {c_{human}}$, $\mathbf {c_{murine}}$ and $\mathbf {c_{porcine}}$.

These prototypes with the mean of the values in each dimensional axis for the species that infect the same host are centroids and can be used to represent all the samples that belong to the corresponding host. So, instead of compute the distances for all the labeled points, the distance to the nearest prototype can be used to deduce the label of the unknown sample.

Let’s say that *D* is the set of distances $d(\mathbf {x},\mathbf {c_{host})}$ from each of the prototypes to the unknown point $\mathbf {x_{new}}$ with $\mathbf {x_{new}}=(x_1,x_2)$:

$$\begin{aligned} D=\{d(\mathbf {x},\mathbf {c_{avian})},d(\mathbf {x},\mathbf {c_{bat})},d(\mathbf {x},\mathbf {c_{bovine})},d(\mathbf {x},\mathbf {c_{human})},d(\mathbf {x},\mathbf {c_{murine})},d(\mathbf {x},\mathbf {c_{porcine})}\}. \end{aligned}$$

We can assume that the label of the unknown point will be the label of the shortest distance in this set:

$$\begin{aligned} host(x)=host(min\ D). \end{aligned}$$

### Modeling, validation and prediction

Since the unknown host sample prediction depends on the distance computation to prototypes, we need to define which metric (distance measure) is more appropriate for our classification problem. In fact, in our case, we are interested in computing the distance between a *z*-dimensional point $\mathbf {x} = (x_1, x_2,\dots , x_z)$ and the centroid $\mathbf {c} = (c_1, c_2,\dots , c_z)$ of a cloud of points representing a given host. Indeed, this distance can be measured by several methods. The most known is the Euclidean distance given by $d_{\text {ED}}(\mathbf {x},\mathbf {c})=\sqrt{(\mathbf {x}-\mathbf {c})(\mathbf {x}-\mathbf {c})^{T}}=\sqrt{\sum _{i=1}^{n}(x_i-c_i)^2}$. But there are other types of distance measures that can reflect more precisely the distribution of the host points in the dataset. One of them is the Mahalanobis distance (MD)^39–41^ which is a measure between a sample point $\mathbf {x} = (x_1, x_2,\dots , x_z)$ and a distribution of sample points represented by:

(i) its centroid $\mathbf {c} = (c_1, c_2,\dots , c_z)$ and
(ii) its covariance matrix *S*.

The distance $d_{\text {MD}}$ is given by $d_{\text {MD}}(\mathbf {x},\mathbf {c})=\sqrt{(\mathbf {x}-\mathbf {c})S^{-1}(\mathbf {x}-\mathbf {c})^T}.$

Taking as an example of our analysis, we will have 74 samples extracted from **bats** and the corresponding 2-dimensional points after the PCA reduction. The centroid of bat will be $\mathbf {c_{bat}} = (c_{1_{bat}},c_{2_{bat}}) = (\bar{x_1}_{bat},\bar{x_2}_{bat}),$ and the covariance matrix $S_{bat}$ for the points that belong to bat will be:

$$\begin{aligned} S_{bat}= \begin{bmatrix} \sigma _{x_{1_{bat}}}^2 &{} \sigma _{x_{1_{bat}}}\sigma _{x_{2_{bat}}}\\ \\ \sigma _{x_{1_{bat}}}\sigma _{x_{2_{bat}}} &{} \sigma _{x_{2_{bat}}}^2 \end{bmatrix}, \end{aligned}$$

where $\sigma _{x_{1_{bat}}}$ is the variance on vertical axis of the points that belong to the host bat and $\sigma _{x_{2_{bat}}}$ is the variance of these points on horizontal axis.

MD is the distance between a point and a set of points in a multivariate space. If the data are non correlated MD it would be as Euclidean distance. It measures distance relative to the centroid, which is the prototype in our case.

MD can improve the accuracy of estimates because it considers correlations between the summary statistics^41^. The use of MD after use PCA exhibit some advantages in terms of computational cost as in the calculation of the covariance matrix *S* and its inverse $S^{-1}$.

The validation is the performance evaluation of the model. This was done with the technique leave-one-out cross-validation in the training dataset (Dataset-1). It consists in removing one point at a time from the dataset to be the unknown point to be predicted and calculating its distance to the grouped points generated according to the host label of the remainig sequences. The shortest distance obtained is considered the prediction and compaired to the known label of the point. If there is a match, it is added to the count to calculate the accuracy. This procedure is repeated to all the sequences of the training dataset. In the end, the relative accuracy can be calculated counting the hits in the prediction. The validation was carried out for several dimensions starting from the reduction of characteristics to two dimensions and increasing until the accuracy reached a level in which there were no relevant changes, which occured in 20-dimension.

All the calculations were made in Python 3.6.9^42^ programming language with Numpy 1.19.5^43^ library , and Biopython 1.19.5^44^ package. Figures 1B,C, 2 and 3 graphics were made with Matplotlib 3.2.2^45^ library.

## Supplementary Information

Supplementary Information 1.

Supplementary Information 2.
